# Does physical activity lower the risk for metabolic syndrome: a longitudinal study of physically active older women

**DOI:** 10.1186/s12877-020-01952-7

**Published:** 2021-01-06

**Authors:** Izabela Zając-Gawlak, Jana Pelclová, Dorota Groffik, Miroslava Přidalová, Agnieszka Nawrat-Szołtysik, Aleksandra Kroemeke, Aleš Gába, Ewa Sadowska-Krępa

**Affiliations:** 1 Institute of Sport Sciences, The Jerzy Kukuczka Academy of Physical Education, Katowice, Mikołowska 72A, 40-065 Katowice, Poland; 2 Faculty of Physical Culture, Palacký University Olomouc, třída Míru 117, 779 00 Olomouc, Czech Republic; 3 Institute of Physioterapy and Health Sciences, The Jerzy Kukuczka Academy of Physical Education, Katowice, Mikołowska 72A, 40-065 Katowice, Poland; 4 Department of Psychology, SWPS University of Social Sciences and Humanities, Chodakowska 19/31, 03-815 Warsaw, Poland

**Keywords:** Health, Daily physical activity, Accelerometer, MetS components, Number of steps

## Abstract

**Background:**

The associations between physical activity and metabolic syndrome (MetS) have been mainly found in cross-sectional studies. The aim of this longitudinal study was to examine the relationship between meeting step-based guidelines and changes in the risk of metabolic syndrome.

**Methods:**

This study included data from older women (baseline age 62.9 ± 4.3 years) from a 7-year longitudinal study in Central Europe. At baseline and follow-up, physical activity was measured by an accelerometer, and the risk for MetS was assessed according to the NCEP-ATP III criteria. In 59 women, multivariate repeated measures ANOVA was used to compare differences in changes in the risk of MetS in groups based on meeting step-based guidelines (10,000 steps/day and 9000 steps/day for women aged <65 and ≥ 65 years, respectively).

**Results:**

Over 7 years, steps/day increased from 10,944 ± 3560 to 11,652 ± 4865, and the risk of MetS decreased from 41 to 12% in our sample. Women who longitudinally met step-based guidelines had a significantly higher mean concentration of high-density cholesterol (HDL-C) (64.5 and 80.3 mg/dL at baseline and follow-up, respectively) and a lower concentration of triglycerides (TGs) (158.3 and 123.8 mg/dL at baseline and follow-up, respectively) at follow-up compared to baseline. Moreover, women who increased their daily steps over 7 years to the recommended steps/day value significantly decreased the concentration of TGs (158.3 mg/dL and 123.8 mg/dL at baseline and follow-up, respectively).

**Conclusions:**

Our study might suggest that the long-term meeting of step-based guidelines or an increase in daily steps/day to achieve the recommended value could be related to a lower risk of MetS, specifically in concentrations of HDL-C and TG. These findings may help in designing interventions aiming to decrease the risk of MetS in older women.

## Background

The concomitance of high blood pressure, hyperglycaemia, central obesity, and elevated cholesterol or triglyceride levels, known as metabolic syndrome (MetS), is indicated as one of the major socioeconomic problems faced by the contemporary world [1]. Research shows that MetS increases the risk of type 2 diabetes mellitus and cardiovascular diseases, and its components are among the main causes of death worldwide [2]. An early and accurate diagnosis of MetS enabling the application of effective therapies can therefore be very instrumental in protecting the health and well-being of populations [1]. This is particularly the case in older adults because the risk for MetS increases with age [3].

There is a growing body of evidence that the risk of developing MetS can be reduced by physical activity [4]. While most authors point to moderate-to-vigorous exercise as being much more effective [5] than light-intensity exercise [4], there is general consent that all attempts to quit a sedentary lifestyle can help prevent the development of MetS [6].

Older people, who are the most sedentary within the population [7] and develop MetS the most often [3, 8], are recommended to choose moderate-to-higher intensity exercise such as walking. As a low-impact and affordable form of exercise requiring neither special skills nor equipment [9], walking is very well suited to the needs and physical capabilities of older people. It also has advantages over other sports and recreational activities in that can be easily included in one’s daily activities and practised at very old ages [9]. It is also of importance that the increasing availability of step-counting technology makes it possible for people to monitor and regulate their daily physical activity [10].

The authors of a recent systematic review found an inverse relationship between the number of steps taken daily and the presence of MetS [4]. In the case of middle-aged and older adults, it is recommended that they take 10,000 steps per day to prevent the development of MetS [11–14].

Strong associations between the daily number of steps and the risk of MetS development are mostly reported by the authors of cross-sectional studies [15]. Relatively few longitudinal studies have thus far been undertaken to determine relationships between physical activity in older adults and age and their risk of MetS [16].

Given the higher prevalence of MetS in women than men [3] and the lack of longitudinal studies investigating changes in MetS related to physical activity, the aim of this study was to examine the relationship between long-term meetings of step-based guidelines (10,000 steps/day and 9000 steps/day for women aged <65 and ≥ 65 years, respectively) and changes in the risk of MetS in older women. Considering that the risk of MetS can be reduced by physical activity [4], we hypothesized that older women longitudinally meeting the step-based guidelines would have a lower risk of MetS.

## Methods

### Design and participants

We analysed the data from a longitudinal study in older women from Central European countries, which has been described elsewhere [17]. One hundred and six physically active women aged 60+ participating in a programme run at the University of Third Age (U3A) were invited to participate in this study. Of those, 51 failed to meet the study inclusion criteria, which required the participants to be able to walk without a prosthetic aid, not use medications for metabolic disorders, and not smoke cigarettes. Therefore, 89 women were assessed during the baseline stage. Over the 7-year period, 2 participants died, 7 had no contact, 5 were ineligible, and 12 refused to participate in the study again.

All the women were informed that their participation was voluntary and that they could withdraw from the study at any time. They provided their written consent for both baseline and follow-up stages.

This study was approved by the Ethics Commission at The Jerzy Kukuczka Academy of Physical Education (reference number. 3/2009). Moreover, an overarching longitudinal study in Central European countries was approved by the Institutional Research Ethics Committee, Faculty of Physical Culture, Palacký University Olomouc (reference number 20/2017).

### Biochemical and anthropometric assessments

Between 8:00 and 10:00 a.m., fasting blood samples were taken from participants, and their systolic/diastolic blood pressure (SDP and DBP, respectively) was measured using a standard mercury sphygmomanometer. The results of two measurements taken at an interval of 15 min were averaged for analysis. The serum concentrations of glucose, high-density lipoprotein cholesterol (HDL-C), and serum triglycerides (TG) were determined using enzymatic assays and commercially available diagnostic kits (Randox UK, cat. no. GL 2623, CH 200, CH 203, TR 1697). Serum was separated in the usual manner and analysed immediately or kept frozen at − 80 °C until analysis.

Waist circumference (WC) was determined to the nearest 0.5 cm using anthropometric tape midway between the lowest rib and the iliac crest in a standing position. The percentages of body fat (PBF) and visceral fat area (VFA) at the umbilical level were determined using an InBody 720 analyser [18, 19] according to the manufacturer’s instructions (Biospace Co., Ltd., Seoul, Korea).

The presence of MetS was determined in line with the NCEP/ATP III revised guidelines [20]. According to the guidelines, MetS occurs when three or more of the following criteria are met: (1) WC ≥88 cm; (2) TG ≥150 mg/dl; 3) HDL-C < 50 mg/dL; 4) systolic blood pressure (SBP) ≥130 mmHg and diastolic blood pressure (DBP) ≥85 mmHg; and 5) fasting glucose level ≥ 100 mg/dl.

### Physical activity assessment

At baseline and follow-up, the participants’ physical activity (PA) was measured using an accelerometer (ActiGraph GT1M, Manufacturing Technology Inc., FL, USA). The accelerometers were worn by the participants in the small pockets of the elastic belts positioned near the right iliac crest for at least 12 h each day over a period of 8 days and were only removed for water exercises and before bedtime. The first day’s readings were excluded from analysis to ensure that the potential reactivity of participants did not compromise the reliability of measurements [21]. All the participants were instructed to record the duration and type of each physical activity they performed during the day (e.g., walking for 10 min).

The time sampling interval of the accelerometers was set at 1 min, an epoch commonly used to measure free-living physical activity (PA) and in epidemiological research [21], and the step mode was activated. The accelerometer readings were processed in ActiLife v6.13.1 (Pensacola, FL, USA).

### Other assessments

Several self-rated data were obtained from the participants. Smoking status and education level were obtained at both time points. At follow-up, the participants were retrospectively asked about their dietary habits over the 7 years and possible treatment for metabolic disorders.

### Data and statistical analysis

The statistical analysis of the data was performed in STATISTICA 12.5 (StatSoft, USA). The descriptive statistics represent means and their 95% confidence intervals. Moreover, the numbers and percentages of women meeting the diagnostic criteria for each MetS component and step-based guidelines were calculated for baseline and follow-up. The number of steps/day was selected as a commonly used indicator of physical activity level in adults and older adults [22–25].

To divide women aged < 65 and ≥ 65 years into physically highly active (meeting step-based guidelines) and physically low active (not meeting step-based guidelines), threshold values of 10,000 and 9000 steps per day, respectively, were used. The first number was adopted from Bassett et al. [15], Freak-Poli et al. [26], Harris et al. [27], and Tudor-Locke et al. [28], who recommend that adults take at least 10,000 steps per day to stay healthy. For women aged 65+, it was lowered to account for the likely effect of their age on their activity.

Based on meeting daily step-based guidelines at baseline and follow-up, women were divided into four physical activity groups. Women in the low-low (LL) group were below the recommended step-based thresholds at both baseline and follow-up. Women in the high-low (HL) group reached their step-based thresholds only at baseline and those in the low-high (LH) group only at follow-up. The high-high (HH) group included women who met step-based guidelines at both time points.

Differences between participants’ anthropometric parameters, MetS components, and physical activity levels measured in 2009 and 2016, as well as between-group differences in the number of steps, were assessed for statistical significance using a paired t-test. The longitudinal changes in MetS components (the effect of time, TE), the between-group differences in MetS components (the effect of physical activity, PAE), and the associations between the groups’ physical activity and changes in MetS components (the interaction effect, INT) were assessed by multivariate repeated-measures ANOVA (MANOVA). The effect size was determined by calculating eta squared (η2) as per the following formula: η2 = SS effect/SS total, where SS effect is the sum of squares for a given effect and SS total is the total of squares for all effects, interactions, and errors [29]. The 95% confidence intervals calculated for individual MetS components were also analysed [30, 31].

## Results

Out of 106 older women, the analytical sample consisted of 59 women with a mean baseline age of 62.9 ± 4.3 years and baseline BMI of 26.8 ± 4.2 kg/m^2^ at baseline, having tertiary (35.6%), secondary (59.2%), and primary (15.2%) education. The women reported no smoking, no change in their dietary habits and no treatment for metabolic disorders for 7 years. Other sample characteristics for baseline and follow-up are shown in Table 1.

**Table 1:** Participants’ characteristics for baseline and follow-up (*N* = 59)

Characteristic	Baseline		Follow-up		Difference
	*M*	95% *CI*	*M*	95% *CI*	%
*Anthropometrics*					
Height (m)	158.2	(156.9, 159.6)	157.3	(155.9, 158.7)	0.6
Weight (kg)	67.1	(64.4, 69.8)	68.0	(65.2, 70.7)	−1.2
BMI (kg/m^2^)	26.8	(25.8, 27.9)	27.7	(26.5, 28.8)	3.2*
VFA (cm^2^)	133.3	(125.2, 141,4)	131.0	(121.6, 140.4)	−1.7
PBF (%)	36.8	(34.9, 38.6)	39.1	(37.2, 40.9)	6.3*
*Metabolic syndrome*					
WC (cm)	84.1	(81.2, 87.1)	85.1	(82.3, 87.9)	1.2
Fasting glucose (mg/dL)	90.3	(86.9, 93.6)	98.8	(95.1, 102.5)	9.4*
HDL-C (mg/dL)	64.4	(60.0, 68.8)	76.5	(71.6, 81.3)	18.8*
TG (mg/dL)	127.5	(115.0, 140.0)	106.3	(94.9, 117.7)	−16.6*
SBP (mmHg)	129.8	(125.7, 133.9)	135.4	(130.3, 140.5)	4.3
DBP (mmHg)	77.0	(74.5, 79.6)	79.9	(77.1, 82.7)	3.8
No. of MetS criteria	2.2	(1.9, 2.6)	1.4	(1.2, 1.7)	−35.6*
Risk of MetS (N, %)	24	40.1	7	11.8	−30.8*
*Physical activity*					
Steps/day	10.944	(10.006, 11.882)	11,652	(10.384, 12.920)	6.5
Steps/day aged < 65 years	11.596	(10.379, 12.814)	12.847	(11.280, 14.414)	10.8
Steps/day aged ≥65 years	9.673	(8.285, 11.060)	9.322	(7.399, 11.244)	−3.3

*M* Mean, *95% CI* Confidence interval, *BMI* Body mass index, *VFA* Visceral fat area, *PBF* Percent body fat, *WC* Waist circumference, *HDL-C* High-density lipoprotein cholesterol, *TG* Triglycerides, *SBP* Systolic blood pressure, *DBP* Diastolic blood pressure, *MetS* Metabolic syndrome

* – statistically significant at *p* < .001

Over 7 years, BMI and PBF significantly increased by 3.2 and 6.3%, respectively, but the risk of MetS decreased from 39 to 12%, and the mean number of criteria for MetS fell by 35.6%. Decreases were also noted in the concentrations of HDL-C and TG, which are MetS risk factors.

**Fig. 1:**
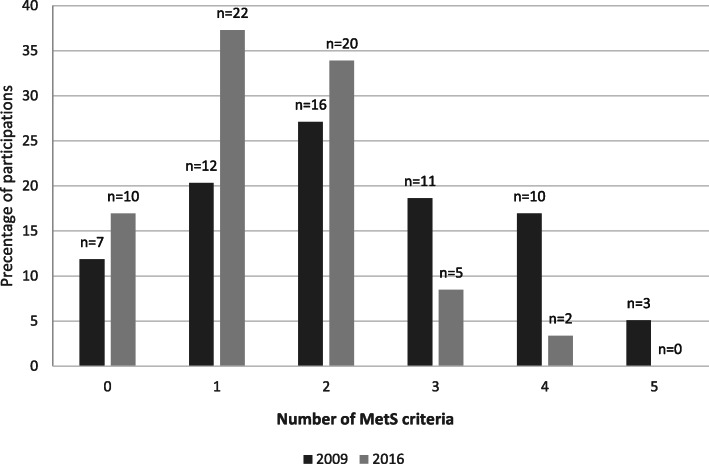
The numbers of MetS criteria met by participants in baseline (2009) and follow-up (2016)

Figure 1 and Table 2 show the range of participants meeting different MetS criteria (0–5). Over 7 years, the number of women who met 3 or more criteria for MetS decreased from 24 to 7. At follow-up, the numbers of participants with elevated TG and BP concentrations and WC ≥ 88 cm decreased by 50, 32.5, and 36.1%, respectively, but the number of women with raised fasting glucose (< 100 mg/dL) increased by 23%. The number of participants with normal HDL-C increased by 58.6%.

**Table 2:** The numbers and percentages of participants (*N* = 59) who met or did not meet particular MetS criteria in baseline and follow-up

MetS component	No. of participants meeting the criterion		No. of participants not meeting the criterion	
	Baseline	Follow-up	Baseline	Follow-up
	*n* (%)	*n* (%)	*n* (%)	*n* (%)
HDL-C	25 (42.4)	1 (1.7)	34 (57.6)	58 (98.3)
TG	16 (27.0)	8 (13.6)	43 (73.0)	51 (86.4)
Fasting glucose	15 (25.5)	23 (39.0)	44 (74.5)	36 (61.0)
BP	40 (68.0)	27 (46.0)	19 (32.0)	32 (54.0)
WC	36 (61.0)	23 (39.0)	23 (39.0)	36 (61.0)

*HDL-C* High-density cholesterol, *TG* Triglycerides, *BP* Blood pressure, *WC* Waist circumference

Long-term differences in steps/day were not significant. However, it is clear from Table 1 that women increased their mean steps/day by 6.5% (10,944 and 11,652 steps/day at baseline and follow-up, respectively). This was mainly due to an increase in daily steps in women aged < 65 (11,596 and 12,847 steps/day at baseline and follow-up, respectively).

Table 3 shows 7-year changes in steps/day in different physical activity groups. Groups LH and HH significantly increased daily steps (*p* = 0.002 and *p* = 0.04, respectively), while the HL group significantly decreased steps/day (*p* = 0.012). No change was found for the LL group.

**Table 3:** Steps/day for different physical activity groups at baseline and follow-up (*N* = 59)

Group^a^	N	Baseline			Follow-up		
		Age	Steps/day		Age	Steps/day	
		*M*	*M*	95% *CI*	*M*	*M*	95% *CI*
LL	12	66.0	7189	(6146, 8231)	73.0	6258	(5068, 7447)
HL	9	64.7	11,182	(9094, 13,269)	71.7	7441*	(5797, 9084)
LH	10	60.8	8005	(7463, 8546)	67.8	13247*	(10,538, 15,957)
HH	28	61.7	13,527	(12,434, 14,621)	68.7	14747*	(13,319, 16,175)

*M* Mean, *95% CI* Confidence interval

* – statistically significant at *p* < 0.05

^a^ *LL* Low-low group (women not meeting step-based guidelines nor in baseline neither in follow-up), *HL* High-low group (women meeting step-based guidelines at baseline but not in follow-up), *LH* Low-high group (women meeting step-based guidelines at follow-up but not in baseline), *HH* High-high group (women meeting step-based guidelines at both time points)

### Changes in physical activity and the risk for MetS

**Fig. 2:**
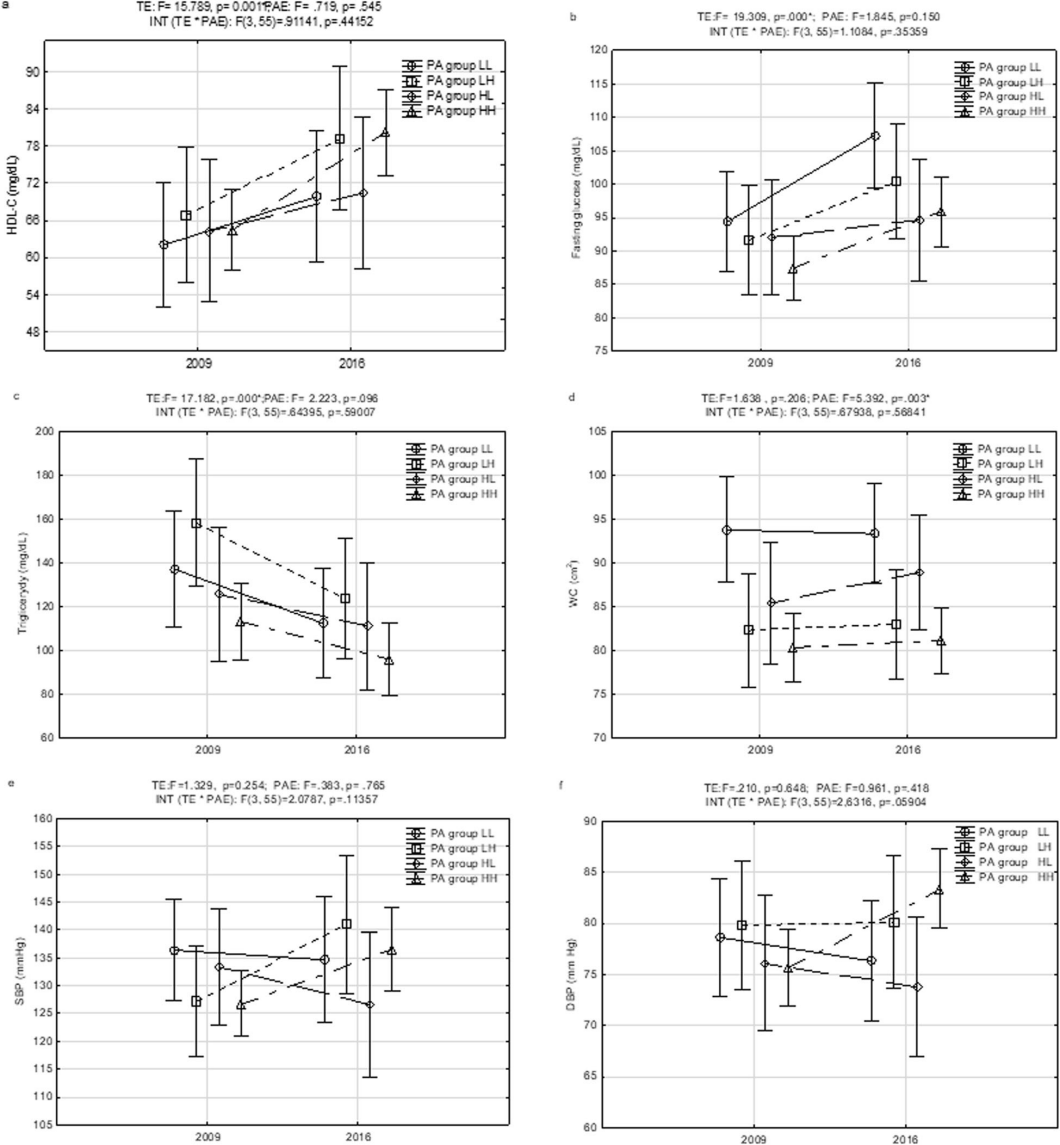
(**a**-**f**) Changes in PA volumes and MetS components between baseline (2009) and follow-up (2016)

According to MANOVA (Fig. 2a-f), significant changes between baseline and follow-up were found for the concentrations of HDL-C (*F* = 15.789, *p* < 0.001), TG (*F* = 17.182, *p* < 0.001) and fasting glucose (*F* = 19.309, *p* < 0.001). The effect of time (TE) was not significant for SBP, DBP, or WC. In follow-up, the HH group and the LH group had significantly higher concentrations of HDL-C than at baseline (64.5 mg/dL [CI: 58.8, 70.2] vs. 80.3 [CI: 73.2, 87.3] and 66.9 [CI: 55.2., 78.6] vs. 79.2 [CI: 73.2, 87.3], respectively). Moreover, the LH group had a significantly decreased concentration of TG (158.3 mg/dL [CI: 105.4, 211.2] vs. 123.8 mg/dL [CI: 82.3, 165.4]). TG decreases in the other three groups were not significant.

All groups had higher levels of fasting glucose in follow-up, but significant changes were noted only in the HH group (87.6 mg/dL [CI: 81.9, 90.3] vs. 95.8 mg/dL [CI: 99.9, 100.8]) and the LL group (94.3 mg/dL [CI: 86.0, 102.7] vs. 107.3 mg/dL [CI: 94.3, 120.3]). The fasting glucose level in the HH group was < 100 mg/dL in both 2009 and 2016.

WC was the only one of the five MetS components that was associated with the levels of participants’ physical activity (*F* = 5.392, *p* = 0.003). At baseline, the HH, LH, and HL groups had smaller mean WCs than the LL group. After 7 years, it was still smaller in the groups that had maintained or increased physical activity (HH and LH), but in the HL group, it increased and was similar to the LL group’s WC.

The interaction effect of time and physical activity was not significant in any of the MetS criteria.

## Discussion

Our longitudinal study in older women suggests a decreasing risk of MetS in longitudinally active older women. Although the effect of group and time was not significant in any of the MetS criteria, women with recommended values of steps/day at baseline and follow-up and women who increased their daily steps over 7 years and met the guidelines in follow-up improved their values in several MetS criteria, mainly in concentrations of HDL-C and TG. Conversely, older women longitudinally not meeting the step-based guidelines had risk values of WC at baseline and follow-up and risk values of fasting glucose at baseline.

Looking at the mean values of steps/day at baseline and follow-up and a 6.5% increase during the 7-year period, our sample consisted of very active women in comparison with the average values of healthy older adults based on normative data [32] and did not support the expected age-related decrease in daily physical activity [33–35]. On the other hand, BMI and PBF significantly increased over 7 years, which is in concordance with other longitudinal studies [36, 37].

It should be mentioned that overweight and obesity may lead to metabolic syndrome after menopause [38–40], especially in older women [41]. In our sample, the risk of MetS decreased over time, although the average values of BMI and PBF were in the overweight (25–29.9 kg/m^2^) and obesity (> 35%) categories at baseline and follow-up. However, abdominal obesity indicators, VFA and WC, which did not change, could be stronger predictors of MetS in older women [14, 42].

In this study, we found improvement in HDL-C and TG in the whole sample, but with the largest change in women in the HH group (longitudinally meeting steps/day guidelines) and in women in the LH group (meeting the guidelines in follow-up). On the other hand, deterioration was found in fasting glucose in the sample, but only women in the LL group (not longitudinally meeting steps/day guidelines) or HL group (not meeting steps guidelines at follow-up) exceeded the risk cut-off point ≥100 mg/dl. WC was the highest in the women longitudinally not meeting the step/day recommendation and in women who decreased their steps/day over 7 years. These findings might confirm previous studies suggesting that the risk values of different MetS components can be reduced by exercise and sufficient physical activity [43–46]. Our findings are in line with the USA study [13], suggesting that adults who took more steps/day tended to have lower WC, higher HDL-C, and lower levels of TG. Additionally, a recent review [4] reported a negative association with MetS in all studies assessing step counts.

The strengths of this study are its longitudinal design with a long follow-up duration and objective assessments of physical activity and MetS criteria. The limitations of the study are mainly the relatively small number of participants impeding the generalization of its results and reducing the power of statistical testing. Moreover, the results of the study were not adjusted for dietary information, although the impact of specific food or products on the risk of MetS was suggested in previous studies [47–49]. Additionally, genetic information, family history or comorbidities that could be related to the risk of MetS were not considered in this study. Another limitation lies in the treatment of physical activity. Steps/day were already used in some previous studies, enabling valuable comparison [4, 13]. However, they did not provide information on the intensity and type of physical activity that might influence the strengths of associations between physical activity and risk of MetS [4].

## Conclusion

Our longitudinal study in a cohort of active older women might suggest that the long-term meeting of step-based guidelines or longitudinal increases in daily steps/day to achieve the recommended value could be related to a lower risk of MetS, specifically in concentrations of HDL-C and TG. These findings may help in designing interventions aiming to decrease the risk of MetS in older women.

## Data Availability

The dataset analyzed during the current study is available in the Figshare repository, https://figshare.com/articles/dataset/Dataset/13234544.

## Abbreviations

MetS: Metabolic syndrome
HDL-C: High-density cholesterol
TG: Triglycerides
VFA: Visceral fat accumulation
BMI: Body mass index
PBF: Percent body fat
BP: Blood pressure
DBP: Diastolic blood pressure
SDP: Systolic blood pressure
WC: Waist circumference
PA: Physical activity
95% CI: Confidence interval
TE: The effect of time
PAE: The effect of physical activity
INT: The interaction effect
TE: The effect of time
LL: Low physical activity at baseline (2009) and follow-up (2016)
HL: High physical activity at baseline (2009) and low at follow-up (2016)
LH: Low physical activity at baseline (2009) and high at follow-up (2016)
HH: High physical activity at baseline (2009) and follow-up (2016)
